# Environmental burden of disease resulting from long-term nitrogen dioxide exposure in Germany

**DOI:** 10.1186/s12889-024-21200-6

**Published:** 2025-01-07

**Authors:** Paulina Sell, Dietrich Plass, Sarah Kienzler, Hajo Zeeb

**Affiliations:** 1 Department for Exposure Assessment and Environmental Health Indicators, German Environment Agency, Berlin, Germany; 2 Department of Prevention and Evaluation, Leibniz Institute for Prevention Research and Epidemiology – BIPS, Bremen, Germany; 3 Health Sciences Bremen, University of Bremen, Bremen, Germany

**Keywords:** Environmental burden of disease, Air pollution, Nitrogen dioxide (NO_2_), Germany, Disability-adjusted life years, Attributable deaths

## Abstract

**Background:**

Exposure to nitrogen dioxide (NO_2_) is associated with an increased risk of cardiovascular, respiratory, and other diseases and health outcomes. Although NO_2_ emissions have decreased in Germany, concentrations currently observed still pose a threat to population health. The aim of this study is to estimate the environmental burden of disease (EBD) resulting from long-term NO_2_ exposure in Germany from 2010 to 2021.

**Methods:**

To estimate the attributable disease burden, World Health Organization’s EBD approach was used. We first conducted a systematic literature review to identify exposure–response functions (ERFs) which mathematically represent the association between NO_2_ exposure and the health outcomes: asthma, chronic obstructive pulmonary disease (COPD), type 2 diabetes mellitus (T2DM), ischemic heart disease, lung cancer, stroke, and cardiovascular and respiratory mortality. Then, we estimated the years of life lost (YLL), years lived with disability (YLD) and, where possible, disability-adjusted life years (DALYs) due to these health outcomes in Germany, using mostly publicly available data. In a third step, using the ERFs and modelled ambient NO_2_ exposure data, the fraction of the disease burden attributable to NO_2_ exposure was estimated for each health outcome and year, stratified by sex.

**Results:**

The systematic review yielded recent ERFs for some exposure-outcome pairs, but not always for both mortality and morbidity outcomes. A full DALY calculation was possible for COPD and T2DM. For the other outcomes, either only YLL or YLD were calculated. Summing up the estimated YLD and YLL of all outcomes, the burden of disease due to NO_2_ exposure in Germany decreased from 261,503 (95% UI 69,290–489,273) lost healthy years in 2010 to 100,032 (95% UI 24,558–191,715) in 2021.

**Conclusions:**

Although the burden of disease attributable to NO_2_ exposure decreased from 2010 to 2021, NO_2_ still poses a threat to population health in Germany. While the current legal concentration limit was generally not exceeded in Germany in 2021, stricter new values proposed by WHO were frequently surpassed. When comparing the results to a previous assessment, it was obvious how strongly different input data impact the results. Transparent reporting of input data and discussing potential challenges when interpreting EBD results are critical.

**Supplementary Information:**

The online version contains supplementary material available at 10.1186/s12889-024-21200-6.

## Background

Since the beginning of the industrialisation and especially the use of diesel-powered engines, nitrogen dioxide (NO_2_) has been posing a threat to the health of populations, particularly in urban areas due to high traffic volume. The European Environment Agency estimated that in Europe, a total of 69,000 deaths (all-natural causes) and 634,721 disability-adjusted life years (DALYs) can be attributed to NO_2_ exposure in 2021 [1]. The highest impact on health was due to diabetes mellitus (314,574 DALYs), stroke (204,723 DALYs), and asthma (115,425 DALYs) in adults. NO_2_ is mainly released into the ambient air through exhaust fumes from motor vehicles and industrial as well as private combustion processes. A downward trend in NO_2_ pollution in Germany has been observed since around 1995 [2] but negative effects on human health can still be observed, even at low levels of exposure [3]. Recent studies suggest that effects of NO_2_ exposure include chronic respiratory and cardiovascular diseases such as chronic obstructive pulmonary disease (COPD) and hypertension [4–6]. Also, increased susceptibility to respiratory infections such as COVID-19 [7–9] and all-cause and cause specific mortality such as ischemic heart disease, lung cancer and stroke mortality [10, 11] was observed in recent studies. People with pre-existing respiratory and cardiovascular conditions, older people and children are particularly vulnerable to the negative effects of NO_2_ exposure, and groups of lower socioeconomic position (SEP) are more strongly affected by air pollution [12–14]. Since the entire global population is potentially affected by polluted ambient air and there are no practical personal protective measures, the relevance of research focusing on the population health effects and disease burden due to NO_2_ exposure is high. Based on the results of respective research projects, political decision-makers can develop guidelines and enact legal regulations to reduce ambient air pollution and effectively protect population health. Currently, the limit values of the European Union (EU) directive 2008/50/EC apply in the EU. They were introduced into German law in 2010. The legal NO_2_ concentration limit is currently set at 40 µg/m^3^ as an annual average concentration. As part of the European Green Deal, the EU Commission proposed a revision of the directive, which should be more closely aligned with the new air quality guidelines of the World Health Organization (WHO) [15]. A limit value of 10 µg/m^3^ NO_2_ annual average was proposed in September 2023 [16]. While the legal limit value of 40 µg/m^3^ was still exceeded at around 75% of measuring stations in Germany in 2010, in 2021 it was only exceeded at around 1% of stations. However, WHO’s proposed maximum NO_2_ concentration of 10 µg/m^3^ was exceeded at 80% of the monitoring stations in 2021 [2].

Given the relatively high NO_2_ exposure of the German population, it is crucial to assess the potential health effects of NO_2_. Therefore, the aim of this study is to quantify the outcome-specific burden of disease attributable to long-term ambient NO_2_ exposure in Germany in the years 2010 to 2021. Currently no up-to date estimates of the disease burden attributable to NO_2_ exposure are available for Germany and thus this study closes a gap for national NO_2_ burden of disease estimations and provides a coherent time series based on enhanced methods and more refined data as compared to previous studies. The study further aims to raise awareness about the current population health effects of NO_2_ in ambient air and helps to establish a foundation for future protective and regulatory actions to protect population health.

## Methods

### Health outcomes and systematic review

The selection of relevant health outcomes associated with NO_2_ exposure was based on a previous environmental burden of disease (EBD) study conducted in Germany, where a systematic review was performed identify outcomes with relevant evidence for an association with NO_2_ [17] (evidence rating by Schneider et al. in brackets): all-cause mortality (weak, including cardiovascular mortality with strong evidence and respiratory mortality with weak evidence), bronchial asthma / bronchitis / COPD (moderate), heart failure / cardiac insufficiency (moderate), type 2 diabetes mellitus (T2DM) (moderate), heart attack (myocardial infarction) (weak), hypertension (moderate), ischemic heart disease (IHD) (moderate), lung cancer (LC) (weak), and stroke (moderate).

Recently published studies on exposure–response functions (ERFs) quantifying these associations for both mortality and morbidity were identified through a systematic literature review, with the initial search conducted on June 28th, 2023, on PubMed utilising adapted search terms from Schneider et al. [17]. The term “AND review* [TIAB]” was added to the original search terms in order to exclusively identify systematic reviews and meta-analyses. Search details can be found in the supplementary materials (Additional file 1). The following inclusion criteria were used: a) Study type: Systematic reviews and meta-analyses; b) ERF reported; c) Long-term effect studied; d) Region: Countries in Europe, USA; e) Outcome of interest (as detailed above) investigated; f) Outdoor ambient NO_2_ studied; g) General population; h) Time period: Published since 1 January 2016; i) Language: English, German. Only systematic reviews and meta-analyses of epidemiological studies were included. Toxicological and animal studies were excluded. Studies that mainly analysed primary studies from regions other than Europe and the USA were not included to ensure applicability of the ERFs to the exposure situation in Germany. The search period was restricted to 1st January 2016 until the date of the search, since the search period of the systematic review by Schneider et al. [17] ended in 2016 and we synthesised the results from both reviews. After title/abstract and full-text screening, the following data was extracted for each study: Digital Object Identifier (DOI), title, year of publication, authors, journal, study type, basis of the study (e.g. cohort), location of study population, study period, outcome, International Statistical Classification of Diseases and Related Health Problems 10th Revision (ICD-10) Code, outcome assessment method, population studied (general or specifically exposed), size of population, population age, sex, NO_2_ exposure assessment, exposure regimen, descriptive measure of exposure, model (single or multi pollutant), statistical model for meta-analysis (random or fixed effects model), confounder, number of effect estimates combined, homogeneity measure, ERF and confidence interval (CI). Furthermore, we applied a series of quality criteria that were chosen from two different sources. We used criteria from Schneider et al. that apply to systematic reviews and meta analyses and added criteria from the Oxford Center for Evidence-Based Medicine (CEBM) [18] (the criteria list can be found in supplementary materials (Additional file 2)). The results were then combined with those of Schneider et al. to provide a comprehensive and up-to-date overview of the evidence. Finally, one ERF most suitable for our setting was selected for each risk-outcome-pair.

### Quantification method

To calculate the health effects of NO_2_ exposure in Germany for the years 2010 to 2021, WHO’s EBD method was applied [19]. Using this method, years lived with disability (YLD), years of life lost (YLL), DALYs, attributable deaths, and attributable cases were estimated for selected diseases separately for each year from 2010 to 2021, stratified by sex. The counterfactual value was set at 10 µg/m^3^ NO_2_, which is based on the maximum annual average concentration proposed in WHO’s updated air quality guidelines [15]. This allows the interpretation that theoretically the estimated disease burden could have been avoided by adhering to the WHO recommendations. The EBD was estimated for the population above 25 years of age, since most of the considered outcomes do not occur at younger ages. No time-discounting or age-weighting were applied. R program version 4.3.2 [20] and RStudio version 2023.9.1.494 [21] were used for all calculations. An exemplary R code containing the basic steps of the calculations can be found in the supplementary materials (Additional file 3).

First, the total burden of disease of each health outcome was calculated, stratified by sex and reference year, using the population size in each five-year age group, life expectancy data, prevalence and mortality data, and disability weights (DWs) for the respective outcomes. Secondly, using exposure data and the selected ERFs (only from single-pollutant models), the population attributable fraction (PAF) for each outcome and year for both mortality and morbidity was calculated using the following formula [22]:

$$\text{PAF = }\frac{{\sum }_{i=0}^{I}{P}_{{e}_{i}}{RR}_{i}-1}{{\sum }_{i=0}^{I}{P}_{{e}_{i}}{RR}_{i}}$$

where P_ei_ is the prevalence of exposure at level i among the total population and RR_i_ is the relative risk (derived from the ERF) at the specific exposure level i. The identified ERFs which provided the risk increase for a health effect were available per 10 ppb NO_2_ or 10 µg/m^3^ NO_2_. In order to harmonise the different units of the concentration increases of the ERFs, we adapted the calculation of the beta values, which are required for further EBD calculation steps and express the increase in risk in a standardised form [17, 23]. In order to compare all ERFs, the calculated beta values can be found in additional file 4. A linear relationship between the exposure and mortality or morbidity risk was assumed. The lower and upper limits of the 95% CI of the RRs were used to estimate the uncertainty interval (95% UI) of the PAF. Further, the uncertainty was propagated to the remaining burden of disease indicators. In case of a lower 95% CI limit below 1, indicating the statistical possibility of a protective effect, the lower limit was set to 1 because protective effects cannot be accommodated in the PAF formula. We conducted a sensitivity analysis by using an alternative ERF for IHD mortality for the exemplary reference year 2021 to assess the potential impact of the ERF on the EBD results. In a third step, the EBD attributable to NO_2_ exposure was estimated by multiplying the PAF by the estimated “crude” burden of disease measures YLL and YLD in each age group, year, and outcome, stratified by sex. DALYs attributable to NO_2_ exposure were calculated by combining attributable YLL and YLD. Attributable deaths and cases were derived by multiplying the number of deaths and cases in all age and sex groups with the respective PAF values.

### Exposure estimation

Population-based exposure data was compiled by the German Environment Agency. Nationwide annual mean NO_2_ rural and urban background concentrations with a spatial resolution of about 2 km × 2 km for the years 2010 to 2021 were modelled using the three-dimensional chemical transport model REM/CALGRID (RCG). In addition, the modelled results were combined with measurement data using the optimal interpolation method [24]. The resulting national NO_2_ annual mean concentrations, stratified by 1 µg/m^3^ concentration classes (N = 49, ranging from 0–1 µg/m^3^ to 48–49 µg/m^3^), were linked to spatial information on population density (100 m × 100 m resolution) from the 2011 census [25]. The population data were scaled for each year according to the official population update. The population was then assigned to the concentration classes.

### Data sources

All data used in this study except NO_2_ exposure data are publicly available. Population size data was obtained from the information system of the German Federal Health Monitoring for the years 2010 to 2021, in five-year age groups, stratified by sex [25]. Information on population density was extrapolated from the most recent census, conducted in 2011. Sex and age-specific life expectancy data was provided by the Federal Statistical Office of Germany (Destatis) and was used for each year in the study period [26]. Cause-specific mortality data was obtained from the German Federal Health Monitoring, and ICD-10 codes were used to define causes of death for each outcome, five-year age group, and by sex. The following ICD-10 Codes were used:

- COPD: J40-J44.9; J47-J47.9
- T2DM: E11-E11.1; E11.3-E11.9 plus the proportion of T2DM from E14
- IHD: I20-I25
- LC: C33-C34.9; D02.1-D02.3; D14.2-D14.3; D38.1
- Stroke: G45-G46.8; I60-I62.9; I63-I63.9; I65-I66.9; I67-I67.3; I67.5-I67.6; I68.1-I68.2; I69-I69.3
- Cardiovascular mortality: I00-I99
- Respiratory mortality: J00-J99

The 12-month prevalence data from the national representative health survey “German Health Update” GEDA 2014/2015-EHIS were used to calculate the prevalence of asthma, COPD, and T2DM up to the year 2017 [27–31]. From 2018 to 2021, prevalence data from GEDA 2019/2020-EHIS were used [32]. Cause-specific DWs were derived from the Global Burden of Disease (GBD) 2019 study and calculated for each sex and age group by dividing the YLD for the respective outcomes in Germany in each sex and age group by the prevalence in each group [33]. An overview of the evidence rating for each outcome, ICD-10 Codes and data sources used can be found in supplementary materials (Additional file 5).

## Results

### Systematic review

**Fig. 1:**
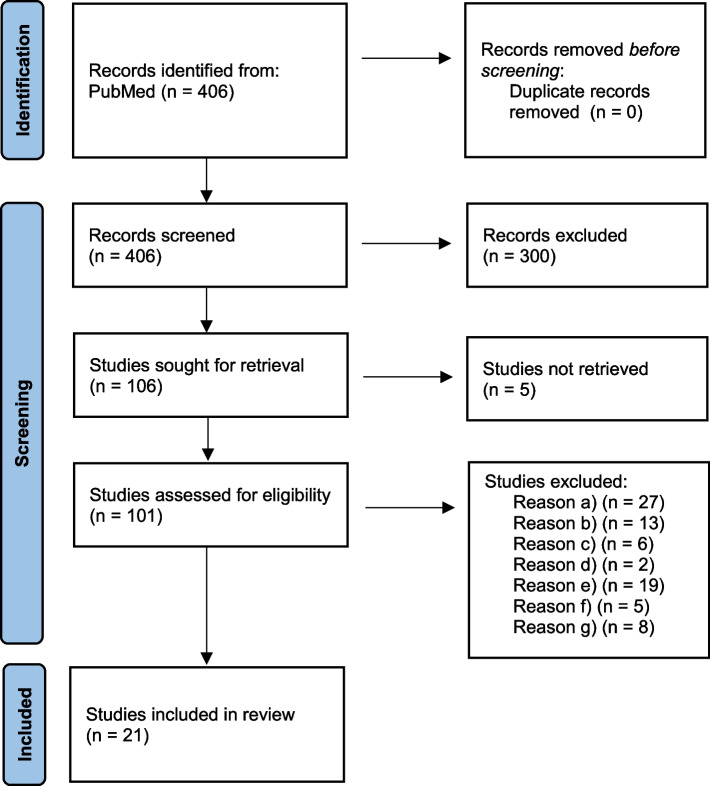
Flow diagram of the systematic review process. Reasons for exclusion (if not fulfilled): **a** Study type: Systematic reviews and meta-analyses; **b** ERF reported; **c** Long-term effect studied; **d** Region: Countries in Europe, USA; **e** Outcome of interest (as detailed above) investigated; **f** Outdoor ambient NO_2_ studied; **g** General population; **h** Time period: Published since 1 January 2016; **i** Language: English, German

The search string used in PubMed identified 406 articles pertaining to the association between the predefined outcomes. 106 articles were selected for full-text eligibility assessment after title and abstract screening, of which 37 articles pertained to all-cause mortality, 17 to bronchial asthma/ bronchitis / COPD, 7 to cardiac insufficiency, 12 to T2DM, 4 to heart attack (myocardial infarction), 9 to hypertension, 4 to IHD, 8 to LC and 8 to stroke. Subsequently, 21 full-text articles were subject to quality assessment (Mortality: 7, bronchial asthma / bronchitis / COPD: 2, cardiac insufficiency: 2, T2DM: 4, heart attack: 0, hypertension: 3, IHD: 0, LC: 0, stroke: 3). An overview of the systematic review process is presented by the flow diagram in Fig. 1.

After quality assessment, 6 studies were rated with low or medium quality and were excluded. A table with an overview of the systematic review process can be found in the supplementary materials (Additional file 2). As the identified meta-analyses for each outcome often contained the same primary studies, pooling of the ERFs was not feasible. Consequently, one ERF was selected for each outcome, according to the criteria of recency, relevance and quality. This was done separately for mortality and morbidity, if available. For T2DM mortality, the ERF from Schneider et al. was adopted, as the systematic review did not yield any new high-quality reviews or meta-analyses on the association with ambient NO_2_ exposure. For stroke mortality, we used an effect measure from the group of cerebrovascular mortality effects. For hypertension mortality as well as IHD and LC morbidity, no suitable ERFs for the association with NO_2_ exposure were found. All identified ERFs were pooled from primary studies using single pollutant models to derive the effect estimate. Table 1 shows the identified ERFs and information on the selected meta-analyses.

**Table 1:** Systematic review results

**Outcome**	**Outcome Type**	**ERF (95% CI)**	**N effect measures combined**	**Heterogeneity (I**^**2**^**)**	**Increment**	**Study**
Cardiovascular	Mortality	HR: 1.14 (1.00, 1.30)	29	99.9%	10 ppb	Stieb et al. 2021 [11]
Respiratory	Mortality	HR: 1.17 (1.02, 1.36)	29	99.7%	10 ppb	Stieb et al. 2021 [11]
Bronchial asthma	Morbidity	OR: 1.26 (1.00, 1.57)	3	n/a	10 µg/m^3^	Schneider et al. 2018 [17]
T2DM	Morbidity	RR: 1.04 (0.96, 1.13)	7	95%	10 µg/m^3^	Kutlar Joss et al. 2023 [34]
T2DM	Mortality	RR: + HR: 1.12 (0.92, 1.36)	2	n/a	10 µg/m^3^	Schneider et al. 2018 [17]
Hypertension	Morbidity	OR: 1.01 (1.00, 1.03)	27	70.1%	10 µg/m^3^	Qin et al. 2021 [5]
IHD	Mortality	HR: 1.13 (1.08, 1.18)	19	96.1%	10 ppb	Stieb et al. 2021 [11]
Stroke	Morbidity	RR: 0.98 (0.92, 1.05)	7	64%	10 µg/m^3^	Haddad et al. 2023 [35]
Cerebrovascular (Stroke)	Mortality	HR: 1.17 (0.94, 1.46)	17	99.7%	10 ppb	Stieb et al. 2021 [11]
Lung cancer	Mortality	HR: 1.08 (1.05, 1.12)	28	96.6%	10 ppb	Stieb et al. 2021 [11]
COPD	Morbidity	HR: 1.07 (1.00, 1.16)	4	82.6%	10 µg/m^3^	Park et al. 2021 [4]
COPD	Mortality	RR: 1.03 (1.01, 1.04)	9	22.7%	10 µg/m^3^	Huangfu & Atkinson 2020 [36]

Identified ERFs and information on studies for the associations of NO_2_ exposure and health outcomes

*CI* Confidence interval, *COPD* Chronic obstructive pulmonary disease, *ERF* Exposure–response function, *HR* Hazard ratio, *IHD* Ischemic heart disease, *OR* Odds ratio, *RR* Relative risk, *T2DM* Type 2 diabetes mellitus

### NO_2_ exposure in Germany

**Fig. 2:**
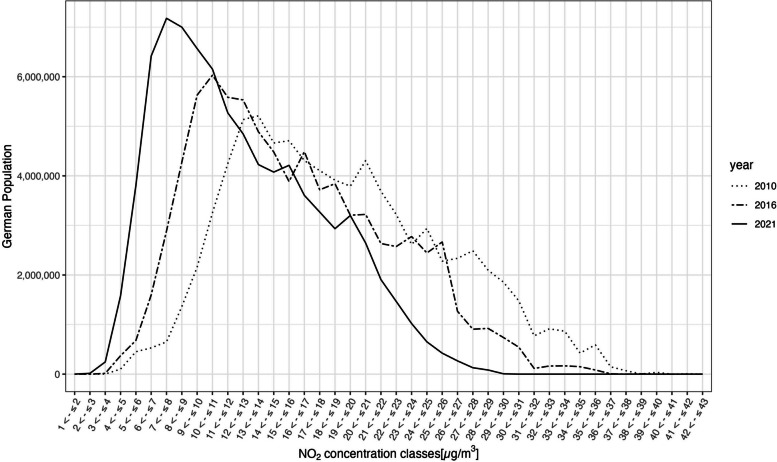
Frequency distribution of the German population in NO_2_ annual mean concentration classes. 2010 (dotted line), 2016 (dashed line), and 2021 (solid line)

A significant shift of the annual mean towards lower urban and rural background NO_2_ concentrations has been observed over the time series from 2010 to 2021. Figure 2 shows an exemplary selection of the NO_2_ exposure years 2010, 2016, and 2021 for comparison. In 2010, the largest share of the German population was exposed to concentrations of about 13-14 µg/m^3^ NO_2_ in ambient air at their residential addresses. By 2016, the level had decreased to 10-11 µg/m^3^ NO_2_ and by 2021, the largest share of the German population was exposed to an annual average of about 7-8 µg/m^3^ NO_2_. According to the available data, 0% of the German population lived in areas exceeding the EU legal annual mean limit values of 40 µg/m^3^. However, in 2021, 60.6% of the German population were exposed to annual NO_2_ background concentrations exceeding 10 µg/m^3^ which corresponds to the recommended maximum annual NO_2_ concentration as suggested in the update of the WHO air quality guidelines [15].

### Environmental burden of disease due to NO_2_ exposure in Germany

**Fig. 3:**
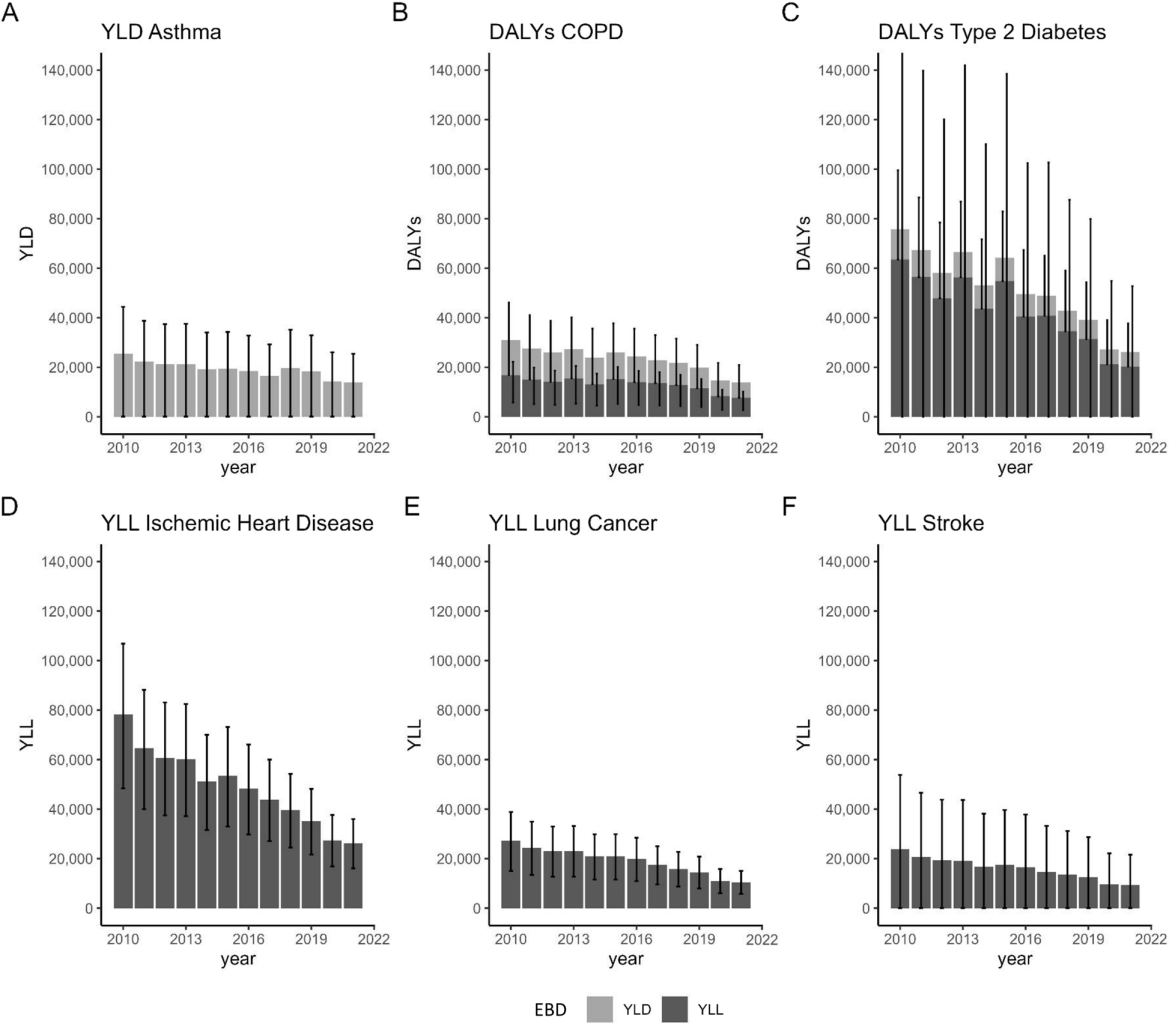
Burden of disease attributable to NO_2_ exposure from 2010 to 2021 including the 95% UI. **A** asthma, **B** COPD, **C** type 2 diabetes mellitus, **D** Ischemic heart disease, **E** lung cancer, and **F** stroke. For T2DM, one 95% UI extends beyond the displayed y-axis range due to scaling. COPD, chronic obstructive pulmonary disease; DALYs, disability-adjusted life years; EBD, environmental burden of disease; UI, uncertainty interval; YLD, years lived with disability; YLL, years of life lost

The EBD for the German population was estimated for the years 2010 to 2021, separately for the outcomes asthma, COPD, T2DM, IHD, LC, and stroke. The EBD due to cardiovascular and respiratory mortality was estimated as an exploratory analysis and should not be added to the disease burden of the specific outcomes (see Fig. 5). A full DALY calculation was only possible for COPD and T2DM, since either the YLD or YLL could not be estimated for asthma, IHD, and LC due to lack of evidence for the respective association. For stroke, a recent meta-analysis [35] showed a protective effect resulting from NO_2_ exposure for stroke incidence. Since the biological plausibility for this association is questionable and protective effects cannot be applied to EBD calculations, the attributable cases and YLD were not calculated and this outcome was excluded from any further analyses. EBD estimations for heart failure / cardiac insufficiency were not feasible due to the high prevalence of inaccurate ICD-10 classification of deaths for this cause [37]. For hypertension, no EBD was calculated because there were no DWs available and no evidence for the association with hypertension mortality was identified in the literature search. For the same reason, no EBD was calculated for heart attack and bronchitis. In Table 2, detailed EBD results for the year 2021 are shown. The complete results table can be downloaded from the supplementary materials (Additional file 6). Summing up all estimated YLL and YLD across all outcomes (excluding cardiovascular and respiratory mortality), a decrease from 261,503 lost healthy years (95% UI 69,290–489,273) in 2010 to 100,032 lost healthy years (95% UI 24,558–191,715) in 2021 is observed, which is mostly attributable to a reduction in NO_2_ exposure in Germany. The highest estimated EBD consistently was related to T2DM and IHD (see Fig. 3).

**Table 2:** EBD results for Germany 2021

**Outcome**		**PAF Mortality**	**PAF Morbidity**	**YLL**	**YLD**	**DALYs**	**Attributable Deaths**	**Attributable Cases**
Bronchial asthma	Both sexes	-	7.17%	-	13,961	-	-	398,859
		-	(0.03%−13.09%)	-	(68–25,472)	-	-	(1,929–727,716)
	Female			-	8,041	-	-	229,399
				-	(39–14,671)	-	-	(1,109–418,537)
	Male			-	5,920	-	-	169,460
				-	(29–10,801)	-	-	(820–309,179)
	Rate per 100,000			-	22.07	-	-	630.45
				-	(0.11–40.26)	-	-	(3.05–1,150.26)
COPD	Both sexes	1.01%	2.28%	7,739	6,216	13,955	625	97,740
		(0.34%−1.34%)	(0.00%−4.84%)	(2,627–10,227)	(0–13,183)	(2,627–23,410)	(212–826)	(0–207,289)
	Female			3,646	3,469	7,114	284	52,291
				(1,238–4,817)	(0–7,356)	(1,238–12,173)	(97–376)	(0–110,899)
	Male			4,093	2,747	6,841	341	45,449
				(1,390–5,409)	(0–5,827)	(1,390–11,236)	(116–450)	(0–96,390)
	Rate per 100,000			12.23	9.83	22.06	0.99	154.49
				(4.15–16.16)	(0–20.84)	(4.15–37.00)	(0.34–1.31)	(0–327.65)
Type 2 diabetes mellitus	Both sexes	3.64%	1.34%	20,340	5,773	26,113	2,776	83,129
		(0.00%−9.44%)	(0.00%−4.03%)	(0–52,794)	(0–17,371)	(0–70,165)	(0–7,206)	(0–250,146)
	Female			11,695	2,669	14,364	1,730	39,279
				(0–30,355)	(0–8,031)	(0–38,387)	(0–4,490)	(0–118,197)
	Male			8,645	3,104	11,749	1,046	43,850
				(0–22,439)	(0–9,340)	(0–31,779)	(0–2,716)	(0–131,950)
	Rate per 100,000			32.15	9.12	41.27	4.39	131.40
				(0–83.45)	(0–27.46)	(0–110.91)	(0–11.39)	(0–395.39)
IHD	Both sexes	2.16%	-	26,181	-	-	2,622	-
		(1.33%−2.97%)	-	(16,093–35,966)	-	-	(1,612–3,603)	-
	Female			9,751	-	-	1,131	-
				(5,994–13,396)	-	-	(695–1,554)	-
	Male			16,430	-	-	1,491	-
				(10,099–22,571)	-	-	(917–2,049)	-
	Rate per 100,000			41.38	-	-	4.15	-
				(25.44–56.85)	-	-	(2.55–5.69)	-
Lung cancer	Both sexes	1.46%	-	10,487	-	-	659	-
		(0.80%−2.10%)	-	(5,771–15,075)	-	-	(363–947)	-
	Female			4,534	-	-	257	-
				(2,495–6,517)	-	-	(142–370)	-
	Male			5,954	-	-	402	-
				(3,276–8,558)	-	-	(221–577)	-
	Rate per 100,000			16.58	-	-	1.04	-
				(9.12–23.83)	-	-	(0.57–1.50)	-
Stroke	Both sexes	2.75%	-	9,335	-	-	852	-
		(0.00%−6.38%)	-	(0–21,627)	-	-	(0–1,974)	-
	Female			4,857	-	-	460	-
				(0–11,254)	-	-	(0–1,066)	-
	Male			4,477	-	-	392	-
				(0–10,373)	-	-	(0–907)	-
	Rate per 100,000			14.75	-	-	1.35	-
				(0–34.18)	-	-	(0–3.12)	-
Sum	Both sexes			74,082 (24,491–135.689)	25,950 (68–56,026)	40,088 (2,627–93,575)	7,534 (2,187–14,556)	579,728 (1,929- 1,185,151)

PAF, YLL, YLD, DALYs, attributable deaths and cases per health outcome and sex, attributable to long-term NO_2_ exposure higher than 10 µg/m^3^ in 2021 (95% UI in brackets). Rate per 100,000 for population above age 25

*COPD* Chronic obstructive pulmonary disease, *DALYs* Disability-adjusted life years, *IHD* Ischemic heart disease, *PAF* Population attributable fraction, *UI* Uncertainty interval, *YLD* Years lived with disability, *YLL* Years of life lost

**Fig. 4:**
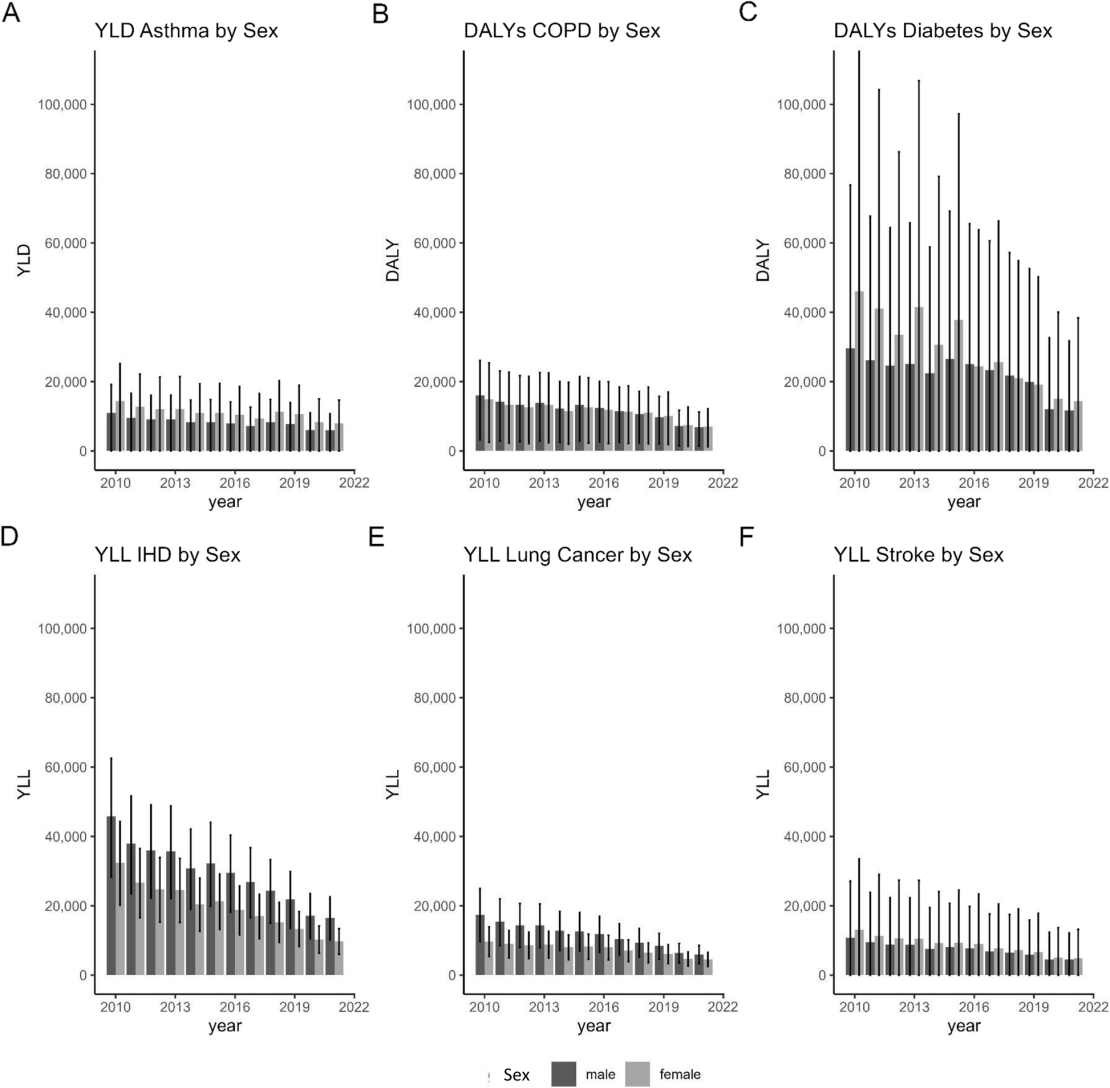
Sex comparison of EBD from 2010 to 2021 including the 95% UI. **A** asthma, **B** COPD, **C** type 2 diabetes mellitus, **D** ischemic heart disease, **E** lung cancer, and **F** stroke. For T2DM, one 95% UI extends beyond the displayed y-axis range due to scaling. COPD, chronic obstructive pulmonary disease; DALY, disability-adjusted life years; EBD, environmental burden of disease; IHD, ischemic heart disease; UI, uncertainty interval; YLD, years lived with disability; YLL, years of life lost

It is important to note that the 95% UI often is very wide, indicating a considerable degree of uncertainty in the EBD estimates. This is due to the width of the 95% CI of the effect measures used. When comparing the disease burden attributable to NO_2_ exposure for females and males (see Fig. 4), it becomes apparent that females were affected by a higher burden resulting from asthma, T2DM, and stroke compared to males. Conversely, males experienced a higher EBD resulting from IHD and LC compared to females.

**Fig. 5:**
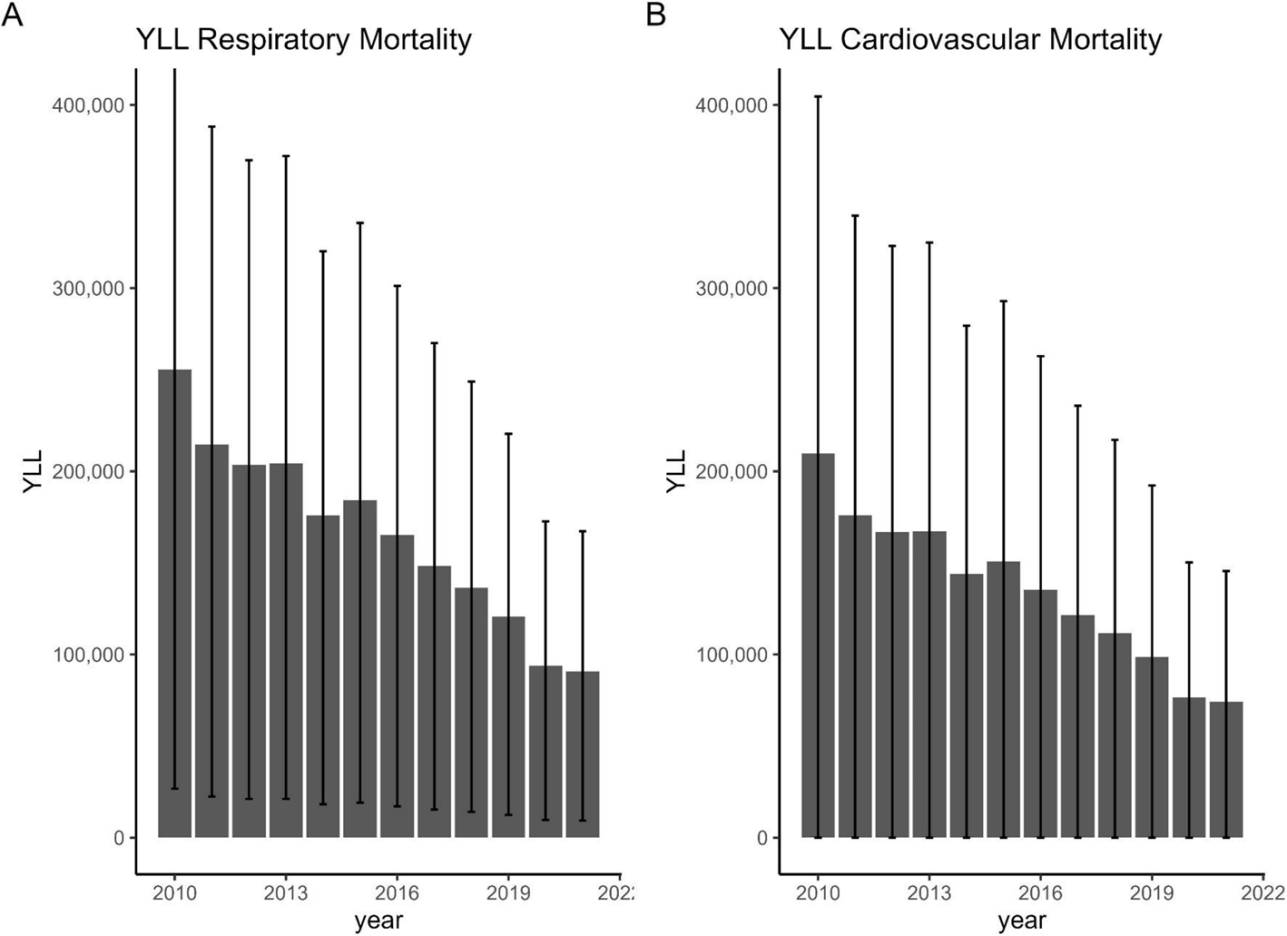
Exploratory analysis: EBD of **A** respiratory and **B** cardiovascular mortality including 95% UI from 2010 to 2021. EBD, environmental burden of disease; UI; uncertainty interval; YLL, years of life lost

The exploratory EBD estimation of the attributable cardiovascular and respiratory mortality in Germany indicated a decreasing trend, comparable to the previously presented results (see Fig. 5). From 2010 to 2021, the EBD for both cardiovascular and respiratory mortality decreased substantially. The estimated EBD due to respiratory mortality was higher than the EBD due to cardiovascular mortality. The 95% UI are very wide, including 0 for cardiovascular mortality, implying very high uncertainty in the EBD estimations.

### Sensitivity analysis

A sensitivity analysis was conducted using an alternative ERF for IHD mortality for the exemplary reference year 2021 to assess the impact of the employed ERF on the EBD results. In their meta-analysis, Stieb et al. [11] reported pooled ERFs with and without exclusion of primary studies with high or probably high risk of bias due to confounding, using BMI rather than smoking as critical potential confounder. In our main analysis, we used the ERFs without exclusion. For this sensitivity analysis, we used the hazard ratio (HR) 1.15 (95% CI: 1.09, 1.21) instead of HR 1.13 (95% CI: 1.08, 1.18) for the association of a 10 ppb increment in NO_2_ concentration and IHD mortality, derived from low or moderate risk of bias studies only.

Results of the sensitivity analysis can be found in Table 3 and can be directly compared to the main results in Table 2.

**Table 3:** Sensitivity analysis

**Outcome**		**PAF Mortality**	**Percent Change**	**YLL**	**Attributable Deaths**	**Percent Change**
IHD	Sum	2.49% (1.61% −3.34 %)	15.28%	30,066 (19,467–40,450)	3,011 (1,950–4,052)	14.84%
	Female			11,198 (7,251–15,066)	1,299 (841–1,748)	
	Male			18,868 (12,217–25,384)	1,712 (1,109–2,304)	
	Rate per 100,000			47.52 (30.77–63.94)	4.76 (3.08–6.40)	

Alternative ERF used for calculation of PAF, YLL, and deaths due to IHD attributable to long-term NO_2_ exposure higher than 10 µg/m^3^ in 2021 (95% UI in brackets) stratified by sex. Rate per 100,000 for population above age 25

*IHD* Ischemic heart disease, *PAF* Population attributable fraction, *UI* Uncertainty interval, *YLL* Years of life lost

As expected, the PAF increased from 2.16% (95% UI 1.33%−2.97%) to 2.49% (95% UI 1.61%−3.34%) and the YLL and attributable deaths increased correspondingly. For the other effect measures pooled by Stieb et al., the HR decreased when excluding high-risk of bias studies. Especially for cerebrovascular (stroke) mortality, the decrease was significant: from 1.17 (95% CI: 0.94, 1.46) to 1.08 (95% CI: 0.97, 1.20).

## Discussion

In this study, the disease-specific EBD attributable to long-term ambient NO_2_ exposure in Germany was estimated for the years 2010 to 2021. Summing up all estimated YLL and YLD across all outcomes (excluding cardiovascular and respiratory mortality), a decrease from 261,503 lost healthy years (95% UI 69,290–489,273) in 2010 to 100,032 lost healthy years (95% UI 24,558–191,715) in 2021 was estimated. We conducted a systematic literature review to identify ERFs for the association of NO_2_ exposure with the selected health outcomes and applied a set of quality criteria. For certain NO_2_ exposure—health outcome pairs (asthma, IHD, LC, and stroke), ERFs for either only morbidity or mortality were identified. For these cases, it was not possible to calculate DALYs; instead, only YLL or YLD could be estimated. For the outcomes hypertension, cardiac insufficiency, heart attack and bronchitis, it was not possible to estimate the EBD due to missing data such as DW or suitable ERFs. For these reasons, the total EBD is likely an underestimate of the true disease burden.

Over the course of the time series, a notable reduction in NO_2_ concentrations was seen in Germany. This decrease is likely caused by the gradual renewal of the vehicle fleet in Germany, as diesel engines are the main source of NO_2_ emissions, and newer engines produce less NO_2_ [38]. Also, there is an increasing number of low-emission vehicles in use in cities. In 2020, NO_2_ emissions also decreased significantly due to the reduced mobility as a result of the COVID-19 pandemic [38]. This steady decrease in exposure most likely led to the decrease in disease burden attributable to NO_2_ exposure. Over half of the attributable disease burden was consistently due to the sum of T2DM morbidity and mortality and IHD mortality. The EBD estimates for asthma, T2DM, and stroke show wide 95% UIs, indicating significant uncertainty. Consequently, these estimates should be interpreted with caution. The sex differences in attributable disease burden (Fig. 4) can be explained by the underlying sex-specific mortality and morbidity patterns; thus, they are not attributable to differences in NO_2_ exposure.

### Comparison with previous EBD estimations for NO_2_ exposure in Germany

We used a similar quantification approach to that of Schneider et al. [17], who estimated the EBD due to NO_2_ exposure in Germany for the years 2007 to 2014. Their study period overlaps with ours in the years 2010 to 2014. Table 4 presents a comparison of the ERFs used in the EBD estimations of both studies. The ERFs used by Schneider et al. differed somewhat from the ERFs used in our study, mainly because they have included both systematic reviews and primary studies and because the meta-analyses that we used to extract the ERFs were not published at the time of the publication of Schneider et al. 2018.

**Table 4:** Comparison of ERFs used in this study with those used by Schneider et al.

**Health Outcome**	**ERF (CI) used in this study**	**RR/HR/OR (CI) used by Schneider et al. **[17]
Asthma Morbidity	OR: 1.26 (1.00, 1.57)	1.26 (1.00, 1.57)
COPD Morbidity	HR: 1.07 (1.00, 1.16)	-
COPD Mortality	RR: 1.03 (1.01, 1.04)	1.05 (0.94, 1.17)
T2DM Morbidity	RR: 1.04 (0.96, 1.13)	1.15 (1.02, 1.29)
T2DM Mortality	RR: 1.12 (0.92, 1.36)	1.12 (0.92, 1.36)
IHD Mortality	HR: 1.13 (1.08, 1.18)^a^	1.06 (1.03, 1.10)
LC Mortality	HR: 1.08 (1.05, 1.12)^a^	-
Stroke Mortality	HR: 1.17 (0.94, 1.46)^a^	1.12 (0.96, 1.31)
Cardiovascular Mortality	HR: 1.14 (1.00, 1.30)^a^	1.03 (1.01, 1.05)
Respiratory Mortality	HR: 1.17 (1.02, 1.36)^a^	-

ERFs per 10 µg/m^3^ increment, including 95% CI

*CI* Confidence interval, *COPD* Chronic obstructive pulmonary disease, *ERF* Exposure–response function, *HR* Hazard ratio, *IHD* Ischemic heart disease, *LC* Lung cancer, *OR* Odds ratio, *RR* Relative risk, *T2DM* Type 2 diabetes mellitus

^a^Per 10 ppb increment

In addition to the different ERFs used, the NO_2_ exposure estimation also differed somewhat. Generally, the same method was used, yet the spatial resolution of 7 km × 8 km was coarser than in our study (2 km × 2 km). As an example, the PAF calculated in our study for COPD was found to be lower than that reported by Schneider et al., since the ERF we used in our estimation was lower (1.03 (95% CI 1.01, 1.04) vs. 1.05 (95% CI 0.94, 1.17) from Schneider et al.). However, the estimated attributable deaths, YLL and YLL per 100,000 were found to be significantly higher in our estimation. This can be explained by higher modelled exposure levels in our assessment, which are a result of the refined spatial resolution of the exposure assessment. The highest NO_2_ concentration class in their data corresponds to 38 µg/m^3^, while in our data, this was 49 µg/m^3^. The PAF for T2DM morbidity was lower in our study, which can be explained by the lower ERF used. Attributable T2DM cases were therefore estimated to be lower in this study compared to Schneider et al. Conversely, the number of YLD due to T2DM was estimated to be higher in our study which can be attributed to the differences in DWs used. While Schneider et al. used values of 0.017 for females and 0.014 for males, we adopted the DWs from the GBD 2019 study [33] with a mean of 0.066 for females and 0.069 for males. Similar considerations apply to differences seen for other outcomes.

### Input data and ERFs used

The preceding discussion shows that the choice of the input data has a major impact on the results of EBD studies [39]. We therefore provide an in-depth discussion of our data choices and the resulting uncertainties.

#### Exposure data

Due to the coarse resolution of NO_2_ annual average concentrations derived from the model, we could only estimate urban and rural background concentrations. Peak concentrations in cities, especially NO_2_ accumulations at roads with high traffic density, were not represented as this would require a much finer resolution. This leads to an underestimation of the NO_2_ exposure and consequently to an underestimation of NO_2_ attributable disease burden. However, the exposure data was calculated on a finer grid than in previous NO_2_ EBD studies in Germany, thereby more accurately representing the true NO_2_ concentrations. It was assumed that the spatial population distribution was constant since 2011, as more recent census data is not available for Germany. However, population size was adjusted in accordance with the official population update for every year of the study period.

#### Morbidity data

Asthma, COPD, and T2DM cases were obtained from the national representative health survey “German Health Update” GEDA 2014/2015-EHIS up to the year 2017. From 2018 to 2021, prevalence rates for self-reported diseases from GEDA 2019/2020-EHIS were used. Especially for asthma and T2DM, a notable increase in prevalence rates is evident from 2017 to 2018. This might be due to real increases in prevalence, but could also result from methodological differences between the two GEDA waves, for example differences in the sample design [40]. Especially for the years more distant from the GEDA prevalence data, the actual number of cases each year might differ from those estimated for this study.

#### Mortality data

Data on deaths due to COPD, T2DM, IHD, and stroke were obtained from the German Federal Health Monitoring and defined by ICD-10 codes. Inaccuracy in IHD death counts may have occurred due to the absence of redistribution of unspecific death codes: Since a large number of deaths in Germany are coded as heart failure, an ill-defined code likely containing many IHD deaths, the death count could be underestimated. For T2DM, this correction was done by including the fraction of T2DM from all diabetes forms from the code group E14, which is classified as “unspecified diabetes mellitus” [37].

#### ERFs used

As clearly shown by the sensitivity analysis, comparing results with the earlier EBD estimations for NO_2_ in Germany and as demonstrated by other studies [41–43], the selection of ERFs has a critical impact on the results. In this study, we aimed to use the most recent ERFs with the strongest evidence for the association between the health outcomes and NO_2_ exposure. Including ERFs only from systematic reviews and meta-analyses in our study has likely led to the non-consideration of recent studies or large pooled cohort studies that were not yet included in systematic reviews and meta-analyses. This is a limitation that needs to be considered when interpreting the results of our study. Some ERFs used in the EBD estimations (T2DM morbidity and mortality, stroke mortality) showed a 95% CI including 1, which means they are not statistically significant. Also, the pooled ERFs often show high levels of heterogeneity. This increases the uncertainty of the results. Nevertheless, we included these exposure–outcome associations in our EBD assessment in order to provide a comprehensive insight into the possible disease burden associated with NO_2_ exposure in Germany. Also, this highlights the need for further research. A linear relationship between NO_2_ exposure and health outcomes was assumed. Although there are some indications for a supralinear relationship with a steeper risk increase at lower concentration levels [44, 45], many studies included in the systematic review found no evidence for a non-linear relationship [46–49]. Overall, there was little investigation into the actual form of the concentration response curves [10, 11]. The sensitivity analysis shows that the EBD results are sensitive towards the choice of ERFs quantifying the association of health outcomes and NO_2_ exposure. A careful and transparent choice of effect measures is therefore important, as are sensitivity analyses.

#### Disability weights

The DWs used in our study were calculated by dividing the annual YLD by the prevalence data for each five-year age group and for both sexes, as available from the GBD 2019 study, since country- and age-specific DWs were not available directly. Using this approach, the DWs were adjusted for severity of the health outcomes and possible co-morbidity. However, if national DWs were available, their use may be preferable, as they are more reflective of the respective target population.

### Social inequity and health effects of NO_2_ exposure

Research has shown that health effects of air pollution in Europe are distributed socially unequal [12, 14]. One reason for this is that individuals with a lower socioeconomic position are disproportionately stronger exposed to air pollution, often residing in neighbourhoods with higher levels of environmental pollution [50]. An inverse relationship has also been observed, where socio-economically advantaged groups living in city centres are exposed to higher levels of air pollution [14, 51]. Bolte et al. point out that low SEP groups have both poorer access to environmental resources (e.g. urban green spaces) and increased vulnerability to the health effects of environmental exposures. “Even if there are no social inequalities in the type and extent of exposure, socially unequally distributed health effects of this exposure can occur due to differences in vulnerability” ([52] p. 675). It is important to take these aspects into account when interpreting the results of this study and when planning and implementing measures to improve air quality. The overall aim should be to protect the whole population from air pollution and thereby improve population health. Since low SEP groups show a greater vulnerability and potentially disease burden due to NO_2_ exposure, they require additional attention.

Due to a lack of data, for example of nationwide small-scale SEP data for differential exposure estimation, a quantitative consideration of SEP in our estimates of the environmental burden of disease (EBD) was not possible. Stratifying the estimations by SEP could provide valuable insights into the links between social inequality and the health effects of air pollution in Germany.

### Interpreting EBD results

The findings of EBD studies should be interpreted with caution and bearing in mind the methodological background and limitations. This is especially important when communicating the insights of such studies to a non-scientific community.

Differences in methods and input data used for EBD studies can lead to significant differences in results, as illustrated in Sect. 3.4. This can limit comparability of different studies. In particular, the decision on which ERFs to use has a strong influence on the results. In this study, we aimed at extracting ERFs from recent meta-analyses with the highest quality, as the field of air pollution research is constantly evolving. Some of the ERFs used are subject to wide uncertainty intervals, which complicate the interpretation of the results. It is therefore crucial to always report both the estimate (e.g. DALYs) with the corresponding uncertainty interval to enable the reader to get a sense for the precision of the results. A limitation that cannot be reported via uncertainty interval pertains for example to the exposure data used to calculate the attributable disease burden. In this study, only annual average background NO_2_ concentrations were modelled. Higher and especially peak concentrations, particularly those in close proximity to busy roads with high surrounding buildings, were not captured in the exposure data, which might lead to an underestimation of the burden of disease. When interpreting and reporting the results, it is important to consider this limitation.

Since we set the counterfactual value to 10 µg/m^3^ NO_2_ in our analyses, which corresponds to the maximum annual average concentration proposed in WHO’s updated air quality guidelines [15], our estimated disease burden can be interpreted as the theoretically avoidable disease burden when adhering to the WHO recommendations. While risk factors such as air pollution are not the direct cause of death, they may contribute to the development of diseases and can be linked statistically to mortality and morbidity on the population level. This is what EBD studies demonstrate and aim to quantify. It is important to emphasise that the results reported in our study are statistical estimates that are not captured in any official medical register. Such analyses provide important information for policy makers and can support environmental and health policy decision making processes.

## Conclusions

Our study shows that NO_2_ pollution in ambient air still poses a relevant threat to population health in Germany, even though the legal concentration limit was mostly not exceeded between 2010 and 2021 and the burden of disease attributable to NO_2_ exposure decreased in the study period. The choice of input data for EBD studies can greatly impact the estimated disease burden attributable to air pollution or other environmental hazards. It is therefore crucial to provide a transparent overview of the input data used and to include a comprehensive discussion of potential methodological limitations.

## Supplementary Information

Supplementary Material 1.

Supplementary Material 2.

Supplementary Material 3.

Supplementary Material 4.

Supplementary Material 5.

Supplementary Material 6.

## Data Availability

Almost all data used in this study are publicly available. The data not publicly available are provided by the corresponding author, PS, upon request.

## Abbreviations

CI: Confidence interval
COPD: Chronic obstructive pulmonary disease
DALY: Disability-adjusted life years
DOI: Digital Object Identifier
DW: Disability weight
EBD: Environmental burden of disease
EU: European Union
ERF: Exposure–response function
GBD: Global Burden of Disease
GEDA: German Health Update
HR: Hazard ratio
ICD: International Classification of Diseases and Related Health Problems
IHD: Ischemic heart disease
LC: Lung cancer
NO_2_: Nitrogen Dioxide
OR: Odds ratio
RR: Relative risk
SEP: Socioeconomic position
T2DM: Type 2 diabetes mellitus
UI: Uncertainty interval
WHO: World Health Organization
YLD: Years lived with disability
YLL: Years of life lost
