# Supplementary material for: Environmental burden of disease resulting from long-term nitrogen dioxide exposure in Germany
Source: BMC Public Health. 2025 Jan 7;25:79. doi: 10.1186/s12889-024-21200-6 (PMC11707916; doi:10.1186/s12889-024-21200-6)
Supplement: Supplementary file 1 — Supplementary Material 1. [file 12889_2024_21200_MOESM1_ESM.pdf]

Additional file 1. Search Details of Systematic Review

**Database**	**PubMed**	
Time Span	2016 until date of search	
Language	English, German	
Species	Human	
**Date of Search**	28.6.2023	
**Outcome**	**Search String**	**N Results**
**Mortality**	mortality[TIAB] AND („nitrogen dioxide“ OR „oxides of nitrogen“ OR „nitrogen oxide” OR „nitrogen oxides” OR NO2 OR NOx) AND review*[TIAB]	102
**Diabetes**	diabetes[TIAB] AND („nitrogen dioxide“ OR „oxides of nitrogen“ OR „nitrogen oxide” OR „nitrogen oxides” OR NO2 OR NOx) AND review*[TIAB]	67
**Hypertension**	(Hypertension[TIAB] OR hypertonia[TIAB] OR „blood pressure“[TIAB]) AND („nitrogen dioxide“ OR „oxides of nitrogen“ OR „nitrogen oxide” OR „nitrogen oxides” OR NO2 OR NOx) NOT (mice[TI] OR mouse[TI] OR rat[TI] OR rats[TI]) AND review*[TIAB]	61
**Cardiac Insufficiency**	(„heart failure” OR „cardiac insufficiency” OR „heart insufficiency” OR „cardiac failure”) AND („nitrogen dioxide“ OR „oxides of nitrogen“ OR „nitrogen oxide” OR „nitrogen oxides” OR NO2 OR NOx) AND review*[TIAB]	26
**Heart Attack**	(infarction[TIAB] OR „heart attack“[TIAB]) AND („nitrogen dioxide“ OR „oxides of nitrogen“ OR „nitrogen oxide” OR „nitrogen oxides” OR NO2 OR NOx) AND review*[TIAB]	14
**Ischemic Heart Disease**	(„ischemic heart disease” OR „ischaemic heart disease” OR „ischemic cardiac disease” OR „ischaemic cardiac disease” OR „coronary artery disease” OR „coronary heart disease” OR „ischemic heart diseases” OR „ischaemic heart diseases” OR „ischemic cardiac diseases” OR „ischaemic cardiac diseases” OR „coronary artery diseases” OR „coronary heart diseases”) AND („nitrogen dioxide“ OR „oxides of nitrogen“ OR „nitrogen oxide” OR „nitrogen oxides” OR NO2 OR NOx) AND review*[TIAB]	16
**Stroke**	(stroke[TIAB] OR apoplexy[TIAB]) AND („nitrogen dioxide“ OR „oxides of nitrogen“ OR „nitrogen oxide” OR „nitrogen oxides” OR NO2 OR NOx) AND review*[TIAB]	27
**Lung Cancer**	(„lung cancer”[TIAB] OR „pulmonary cancer”[TIAB] OR „bronchial carcinoma”[TIAB]) AND („nitrogen dioxide“ OR „oxides of nitrogen“ OR „nitrogen oxide” OR „nitrogen oxides” OR NO2 OR NOx) AND review*[TIAB]	23
**Asthma / Bronchitis / COPD**	(asthma[TIAB] OR bronchitis[TIAB] OR „chest cold”[TIAB] OR bronchiolitis[TIAB] OR „chronic obstructive”[TIAB] OR COPD[TIAB] OR COAD[TIAB]) AND („nitrogen dioxide“ OR „oxides of nitrogen“ OR „nitrogen oxide” OR „nitrogen oxides” OR NO2 OR NOx) NOT (mice[TI] OR mouse[TI] OR rat[TI] OR rats[TI] OR murine[TI]) AND review*[TIAB]	70
**Sum**		406

Detailed information on database, filters and search strings used and number of results.
