# Supplementary material for: Environmental burden of disease resulting from long-term nitrogen dioxide exposure in Germany
Source: BMC Public Health. 2025 Jan 7;25:79. doi: 10.1186/s12889-024-21200-6 (PMC11707916; doi:10.1186/s12889-024-21200-6)
Supplement: Supplementary file 4 — Supplementary Material 4. [file 12889_2024_21200_MOESM4_ESM.pdf]

Additional file 4. ERFs and Beta Values Identified in Systematic Review and used by Schneider et al. 2018

Outcome	Outcome Type	ERFs (95 % CI) identified in systematic review	Calculated ß values (95 % CI)	ERF (95 % CI) used by Schneider et al.	Calculated ß values (95 % CI)
Cardiovascular	Mortality	1.14 (1.00, 1.30)*	0.007 (0, 0.014)	1.03 (1.01, 1.05)	0.003 (0.001, 0.005)
Respiratory	Mortality	1.17 (1.02, 1.36)*	0.008 (0.001, 0.016)		
Bronchial asthma	Morbidity	1.26 (1.00, 1.57)	0.023 (0, 0.045)	1.26 (1.00, 1.57)	0.023 (0, 0.045)
T2DM	Morbidity	1.04 (0.96, 1.13)	0.004 (-0.004, 0.012)	1.15 (1.02, 1.29)	0.014 (0.002, 0.025)
T2DM	Mortality	1.12 (0.92, 1.36)	0.011 (-0.008, 0.03)	1.12 (0.92, 1.36)	0.011 (-0.008, 0.031)
Hypertension	Morbidity	1.01 (1.00, 1.03)	0.001 (0, 0.003)		
IHD	Mortality	1.13 (1.08, 1.18)*	0.007 (0.004, 0.009)	1.06 (1.03, 1.10)	0.006 (0.003, 0.01)
Stroke	Morbidity	0.98 (0.92, 1.05)	-0.002 (-0.008, 0.005)		
Cerebrovascular (Stroke)	Mortality	1.17 (0.94, 1.46)*	0.008 (-0.003, 0.02)	1.12 (0.96, 1.31)	0.011 (-0.004, 0.027)
Lung cancer	Mortality	1.08 (1.05, 1.12)*	0.004 (0.003, 0.006)		
COPD	Morbidity	1.07 (1.00, 1.16)	0.007 (0, 0.015)		
COPD	Mortality	1.03 (1.01, 1.04)	0.003 (0.001, 0.004)	1.05 (0.94, 1.17)	0.005 (-0.006, 0.016)

ERFs per 10 µg/m^3 increment in NO_2 concentration. *per 10 ppb increment. Calculated beta (ß) values for comparing ERFs with different increments (µg/m^3 vs. ppb). CI, Confidence interval; COPD, chronic obstructive pulmonary disease; ERF, exposure-response function; HR, hazard ratio; IHD, ischemic heart disease; OR, odds ratio; RR, relative risk; T2DM, type 2 diabetes mellitus.
