# Supplementary material for: Environmental burden of disease resulting from long-term nitrogen dioxide exposure in Germany
Source: BMC Public Health. 2025 Jan 7;25:79. doi: 10.1186/s12889-024-21200-6 (PMC11707916; doi:10.1186/s12889-024-21200-6)
Supplement: Supplementary file 5 — Supplementary Material 5. [file 12889_2024_21200_MOESM5_ESM.pdf]

Additional file 5. Evidence Rating and Data Sources

Outcome	Evidence Rating by Schneider et al. 2018	ICD-10 Code (for mortality data)	Prevalence Data Reference	Disability Weight Reference
Asthma	moderate		GEDA 2014/2015-EHIS, GEDA 2019/2020-EHIS	Global Burden of Disease (GBD) 2019
Type 2 Diabetes Mellitus	moderate	E11-E11.1; E11.3-E11.9 plus the proportion of T2DM from E14	GEDA 2014/2015-EHIS, GEDA 2019/2020-EHIS	Global Burden of Disease (GBD) 2019
Hypertension	moderate			
Ischemic Heart Disease	moderate	I20-I25		
Stroke	moderate	G45-G46,8; I60-I62,9; I63-I63,9; I65-I66,9; I67,0-I67,3; I67,5-I67,6; I68,1-I68,2; I69,0-I69,3		
Lung Cancer	weak	C33-C34.9; D02.1-D02.3; D14.2-D14.3; D38.1		
Chronic Obstructive Pulmonary Disease	moderate	J40-J44.9; J47-J47.9	GEDA 2014/2015-EHIS, GEDA 2019/2020-EHIS	Global Burden of Disease (GBD) 2019
Cardiovascular mortality	strong	I00-I99		
Respiratory mortality	weak	J00-J99		

Overview of the evidence rating for each outcome, ICD-10 Codes and data sources. GEDA, "German Health Update" survey; ICD-10, International Statistical Classification of Diseases and Related Health Problems 10th Revision, T2DM, type 2 diabetes mellitus.
